# Does quality of care for hypertension in primary care vary with postcode area deprivation? An observational study

**DOI:** 10.1186/1472-6963-11-297

**Published:** 2011-11-02

**Authors:** Salah Hammouche, Richard Holland, Nicholas Steel

**Affiliations:** 1 School of Medicine, Health Policy and Practice, University of East Anglia, Norwich, NR4 7TJ, UK

**Keywords:** Hypertension, Inequalities, Primary Health Care, Quality Indicators

## Abstract

**Background:**

Hypertension is a common major risk factor for stroke and coronary heart disease. Little is known about how achievement of financially incentivised and non-incentivised indicators of quality of care varies with deprivation, or about the effect of financial incentives on health inequalities in hypertension. General practices in the UK have received financial incentives for high quality care since 2004. This study set out to assess the variations in achievement of incentivised and non-incentivised quality indicators for hypertension by patient area deprivation, before and after the introduction of financial incentives.

**Methods:**

Achievement of 14 quality indicators for hypertension in 304 patient participants in 18 general practices in Norfolk, England was assessed one year before (2003) and one year after (2005) the introduction of financial incentives. Four indicators were incentivised and 10 were non-incentivised. Each participant's postcode was linked to an index of multiple deprivation score.

**Results:**

The range of achievement of incentivised quality indicators was 65-94% in the least deprived third of participants, and 77-94% in the most deprived third in 2003 and 2005 combined. For non-incentivised indicators, the range was 7-85% in the least deprived and 24-93% in the most deprived third.

Achievement of incentivised quality indicators in 2003 and 2005 combined did not vary significantly by area deprivation. Achievement of three of 10 non-incentivised indicators was higher in participants from more deprived postcode areas: providing lifestyle advice (odds ratio 1.34, 95% confidence interval 1.00-1.79), assessment of peripheral vascular disease (1.54, 1.02-2.35) and electrocardiography (1.38, 1.04-1.82).

**Conclusions:**

Participants from more deprived areas received at least the same, and sometimes better, quality of care than those from less deprived areas. Quality of care for hypertension in general practice may not follow the inequitable distribution seen with some other conditions.

## Background

Almost 40% of the adult U.K. population have hypertension [1]. Anti-hypertensive medication accounts for 15% of the total annual cost of all primary care prescriptions, with the state-run NHS (National Health Service) funding more than 90 million prescriptions annually [2]. Hypertension is an important risk factor for stroke and coronary heart disease [3,4]. High quality care for hypertension in primary care settings can minimise these adverse effects [4,5].

The General Medical Services contract was revised in 2004. Since then, general practices in England and Wales have been financially rewarded for achievement of quality indicators in at least 10 chronic conditions set out in the Quality and Outcomes Framework [6]. Five quality indicators were for hypertension management.

Previous studies of the Quality and Outcomes Framework (QOF) have reported small inequalities in the achievement of quality indicators by practice area deprivation, with conflicting findings about the direction of inequalities. Studies that used the national database reported lowest achievement in the most deprived areas [7,8]. A regional study reported no evidence of socioeconomic inequality [9]. All these studies used the national QOF database rather than collecting data for each patient individually. None of these studies assessed non-incentivised aspects of primary health care in hypertension; they evaluated the quality indicators that are financially rewarded by the general medical service contract [7-9]. They measured deprivation at the level of general practices, rather than patient postcodes.

This study was set out to assess whether achievement of incentivised and non-incentivised quality indicators for hypertension varied with patients' postcode area deprivation score, and to compare variations one year before and one year after the introduction of the General Medical Service contract in 2004.

## Methods

Eighteen general practices in Norfolk Primary Care Trust were selected to give equal numbers of patients in each of three deprivation groups stratified by national deprivation score. These practices represented about 12% of the total number of practices in the trust [10]. The selected practices represented a wide range of different sizes (2,700 to 17,700 patients).

We assessed the quality of care for hypertension treatment by obtaining the recorded achievement of four indicators incentivised in the Quality and Outcomes Framework (QOF), and ten non-incentivised indicators. All quality indicators used were evidence-based and peer-reviewed, and came from at least one of four sources: the 2004 QOF [6], the National Institute for Health and Clinical Excellence (NICE) [11], Quality Indicators for General Practice (QIGP) developed at the National Primary Care Research and Development Centre [12], or RAND Health indicators adapted for the UK (QSHC) [13]. Achievement data reflected recorded achievement in the medical records, and included any patients who might have been reported as exceptions under the quality and outcome framework. The indicators used in this study are shown in Table 1.

**Table 1:** Quality Indicators

**Incentivised quality indicators from Quality and Outcome Framework (QOF) **[6]		
**QOF 1**	The percentage of patients with hypertension whose notes record smoking status at least once	

**QOF 2**	The percentage of patients with hypertension who smoke, whose notes contain a record that smoking cessation advice has been offered at least once	

**QOF 3**	The percentage of patients with hypertension in which there is a record of blood pressure in the last 9 months	

**QOF 4**	The percentage of patients with hypertension in whom the last blood pressure (measured in the last 9 months) is 150/90 or less	

**Non-incentivised quality indicators**		Source

**NonQOF 1**	The percentage of patients with hypertension who have been recommended lifestyle modification for treatment of hypertension at least once	NICE [11] QSHC [13]

**NonQOF 2**	The percentage of patients with hypertension whose notes document assessment of the following within three months prior to diagnosis: • Personal history of peripheral vascular disease	NICE [11]

**NonQOF 3**	• Diabetes	

**NonQOF 4**	• Hyperlipidaemia	QIGP [12]

**NonQOF 5**	• Alcohol consumption (at any time up to relevant assessment cut-off date)	

**NonQOF 6**	The percentage of patients with hypertension whose notes document the following laboratory investigations and tests from within three months prior to diagnosis: • Urine strip test for protein	NICE [11]

**NonQOF 7**	• Serum creatinine and electrolytes	

**NonQOF 8**	• Blood glucose	

**NonQOF 9**	• Serum/total cholesterol	QIGP [12]

**NonQOF 10**	• ECG (at any time up to relevant assessment cut-off date)	

### Data Collection

This study was a part of a larger study to assess the impact of incentive payments on general practice performance one year before (2004) and one year after (2005) the introduction of the general medical service contract in 2004 [14]. The patient inclusion criteria were any patient aged 18 or over, with diagnosed hypertension and at least one record of systolic BP > 150 or diastolic BP > 90, prior to 1 April 2003 and 2005 respectively. Data were extracted from the patients' electronic and paper records. Sampling from 2003 and 2005 was conducted independently. Between 20 and 40 randomly selected eligible patients at each practice were contacted for permission to examine their full records. All participants gave informed consent. Patient's age, gender, smoking status, year of diagnosis and postcodes were retrieved from the notes.

### Patient and Practice deprivation calculation

The Office for National Statistics (ONS) determined the Index of Multiple Deprivation (IMD) in 2004 [15] by combining a number of indicators chosen to cover a range of economic, social and housing issues into a single deprivation score for each small area. These small areas are called LSOA (Lower Super Output Area), of which there are 32,482 in England [16]. Each LSOA consists of about 1,500 people within a defined geographical locality. This allows each area to be ranked relative to one another according to their level of deprivation [15]. Nationally IMD scores for each LSOA vary from 0.37 in the least deprived area to 85.46 in the most deprived one.

Each patient's index of multiple deprivation (IMD) 2004 score was determined by mapping his/her postcode to the relevant LSOA [15,16]. Patients were categorised into three groups according to their IMD scores in comparison to the rest of the sample. To give almost equal numbers in each group, patients with IMD scores of less than 11.6 were grouped in the least deprived group, those between 11.6 and 18.9 in the middle group and those with an IMD of 19 or more in the most deprived group.

The practice deprivation score was the weighted mean of the IMDs for the postcodes of all registered patients. Practice deprivation scores ranged from 8%-85% of the full range of national practice deprivation scores. Practices were classified according to the national deprivation rank for general practices into three groups. The practices with a total deprivation rank below 30% of the national deprivation rank were classed as most deprived and those above 65% were classed as least deprived.

### Data Analysis

The primary outcome measurement for each participant was the achievement of each quality indicator with a binary (yes/no) score. For each indicator, the percentage of eligible patients for whom the indicator was achieved was calculated.

Associations between indicator achievement and patient and practice characteristics were evaluated using logistic regression models. The outcome variable was quality indicator achievement, and the explanatory variables were patient and practice characteristics. Patient characteristics were: age group (< 63 years old, 63-73, or > 73), gender, smoking status, deprivation group, time point (2003 or 2005), year of diagnosis (1967-1997 or 1998-2005). Practice characteristics were practice size and deprivation group. If 5,000 patients or less were registered in the practice, it was classed as small. Those practices with more than 10,000 patients were classed as large practices.

Firstly, we performed univariate analyses for each quality indicator against patient and practice characteristics separately. We also carried out a subgroup deprivation analysis by time; dividing the sample into two groups (2003, 2005). Secondly, we did a multivariate analysis for each indicator including all possible explanatory variables, we eliminated in a stepwise fashion those variables that were not statistically significant, until only statistically significant (p < 0.05) exposure variables remained in the model. Data analyses were conducting using STATA (Version 8 SE, Texas US).

## Results

A total of 399 patients were invited to participate. Three hundred and four patients (76%) of the invited group agreed to participate and their records were assessed. The mean age of participants was 67 years (SD = 11.9), and 59% were females, compared with a mean age of 67 years and 58% females in the whole population invited to participate. Sample characteristics are provided in Table 2. The number of participants eligible for each indicator is shown in Table 3.

**Table 2:** Sample characteristics

Patient characteristics	Mean (SD), range	Category	N (%)
**Age**	67.4 (11.9), 32-92	< 63 years	101 (33.2)
		63-73 years	101 (33.2)
		> 73 years	102 (33.6)

**Gender**		Men	126 (41.5)
		Women	178 (58.6)

**Smoking status** (n = 280)*		Smoker	42 (15.0)
		Non-Smoker	238 (85.0)

**Time point**		2003	149 (49.0)
		2005	155 (51.0)

**Year of diagnosis**		1967-1997	152 (50)
		1998-2005	152 (50)

**Deprivation score (IMD**)**	19.5 (14.2), 2.6-73.9	Least deprived	102 (33.9)
		Middle	99 (32.9)
		Most deprived	100 (33.2)

**Practice characteristics**			

**Size (patients)**	9610 (4178), 2,700-17,000	Small (2,700-5,000)	47 (15.5)
		Medium (5,400-10,000)	152 (50.0)
		Large (10,500-17,700)	105 (34.5)

**Deprivation score (%)**	48.0 (24.3), 7.5-84.9	Least deprived	114 (37.5)
		Middle	97 (32.0)
		Most deprived	93 (30.6)

Total number of participates was 304

* Smoking status missing for 24 participants

** IMD missing for 3 participants.

**Table 3:** Percentage of participants who achieved each quality indicator, overall, and by gender, age group, time, and practice size.

Quality Indicator	*No*	*Overall achievement N (%)*	*Gender* *(%)*		*Age group* *(%)*			*Time point* *(%)*		*^#^Practice size* *(%)*		
						
			*Men*	*Women*	*< 63 years*	*63-73 years*	*> 73 years*	*2003*	*2005*	*S*	*M*	*L*
**QOF 1** Smoking status	302	227 (91.7%)	94	90	89	91	95	84**	99**	94	90	92

**QOF 2** Smoking cessation advice	42	33 (78.6%)	84	70	86	80	60	53**	96**	89	78	73

**QOF 3** **Record of **BP	303	285 (94%)	95	93	93	96	93	93	95	89	95	94

**QOF 4** BP < 150/90	286	196 (68.5%)	72	66	67	72	66	58***	79***	64	67	72

**Non-QOF 1** Lifestyle advice	304	187 (61.5%)	66	58	64	59	60	54**	59**	72	60	59

**Non-QOF 2** PVD	304	51 (16.8%)	24**	11**	7***	16***	27***	15	19	38***	15***	10***

**Non-QOF 3** DM	304	74 (24.3%)	32*	19*	21	22	30	20	28	21	25	25

**Non-QOF 4** Hyperlipidaemea	304	143 (47%)	52	44	45	55	41	41	52	49	42	53

**Non-QOF 5** Alcohol consumption	303	223 (73.6%)	78	70	69	73	78	68*	79*	85	72	70

**Non-QOF 6** Protein in urine	304	187 (61.5%)	58	64	53**	59**	72**	61	62	79	59	58

**Non-QOF 7** creatinine and electrolyte	304	274 (90.1%)	90	90	91	87	92	85**	95**	91	88	93

**Non-QOF 8** Blood glucose	302	209 (69.21%)	74	66	64	73	71	62*	76*	67	72	66

**Non-QOF 9** Serum/total cholesterol	302	241 (79.8%)	83	77	79	87	73	72**	88**	76	81	80

**Non-QOF 10** ECG	304	152 (50%)	51	49	51	42	57	44	55	49	47	54

^#^Practice size:

**S**: 5.000 patients or less

**M**: 5.001-9.999 patients

**L**: 10.000 patients or more

Univariate logistic regression analysis:

* p < 0.05

** p < 0.01

*** p < 0.001

### Association between deprivation and achievement-univariate analysis

The results were similar whether deprivation was derived from patient postcode area deprivation or from practice deprivation. All results presented below refer to patient postcode area deprivation rather than practice deprivation.

Overall achievement of incentivised quality indicators at patient level did not vary significantly by geographic deprivation (univariate logistic regression analysis) (Table 4). Achievement of incentivised indicators ranged from 65% of participants in the least deprived third for indicator QOF 4 (blood pressure control), to 94% for QOF 3 (blood pressure recorded). The range for the same indicators for participants in the most deprived third was 77% (QOF 4) to 94% (QOF 3).

**Table 4:** Percentage of participants in each deprivation group who achieved each quality indicator, overall and at two time points.

Quality Indicator	Achievement (%)								
			
	All			2003 only			2005 only		
	**Least** **deprived**	**Middle**	**Most** **deprived**	**Least** **deprived**	**Middle**	**Most** **deprived**	**Least** **deprived**	**Middle**	**Most** **deprived**

**QOF 1** Smoking status	94	90	92	87	82	83	100	97	100

**QOF 2** Smoking cessation advice	90	67	80	---*	---*	---*	---*	---*	----*

**QOF 3** Record of BP	94	94	94	91	92	96	96	96	92

**QOF 4** BP < 150/90	65	65	77	***71***	***75***	***84***	87	82	89

**NonQOF 1** Lifestyle advice	56	60	70	51	49	60	60	71	77

**NonQOF 2** PVD	***7***	***20***	***24***	6	17	20	***7***	***23***	***27***

**NonQOF 3** DM	19	30	25	21	25	15	***16***	***35***	***35***

**NonQOF 4** Hyperlipidaemea	40	47	52	36	43	42	44	52	62

**NonQOF 5** Alcohol consumption	71	77	74	66	72	66	75	81	83

**NonQOF 6** Protein in urine	60	63	62	62	65	56	58	60	67

**NonQOF 7** creatinine & electrolyte	85	91	93	***72***	***90***	***92***	96	94	94

**NonQOF 8** Blood glucose	70	74	66	66	63	62	73	85	71

**NonQOF 9** Serum/total cholesterol	78	79	83	70	73	73	85	85	92

**NonQOF 10** ECG	***40***	***55***	***56***	***30***	***49***	***54***	49	60	58

---*: subgroup analysis was not performed due to the small number of eligible patients.

Statistically significant results (p < 0.05) shown in ***bold and italic ***(univariate regression analysis).

For non-incentivised quality indicators, the achievement of indicators did not vary significantly by deprivation for 8 out of 10 indicators. For the remaining two indicators, achievement was significantly higher in the more deprived areas. Achievement ranged from 7% to 85% in the least deprived third, compared to 24%-93% in the most deprived third.

Subgroup univariate analysis was then performed grouping patients according to time point into 2003 or 2005 group. For incentivised indicators in 2003, there was no difference in achievement of 3 out of 4 indicators by area deprivation, with the remaining one indicator showing significantly higher achievement in more deprived groups. In 2005 there were no differences by deprivation for incentivised indicators. For non-incentivised indicators, achievement of 2 of 10 indicators was significantly higher in more deprived groups in both 2003 and 2005.

### Association between deprivation and achievement after adjustment for other factors-multivariate analysis

Achievement of incentivised indicators did not vary by patient area deprivation (Table 5). For non-incentivised indicators, there was statistically significant variation in achievement of three quality indicators by deprivation. The achievement was higher in the most deprived group in providing lifestyle advice (odds ratio 1.34, 95% confidence interval 1.00-1.79), assessment of peripheral vascular disease (1.54, 1.02-2.35) and electrocardiography (1.38, 1.04-1.82).

**Table 5:** Odds ratios for achievement of quality indicators by exposure variables: Multivariate stepwise regression analysis.

Quality Indicator	Gender Women vs. Men	Age (> 73) vs. (73-63) vs. (< 63)	Time point 2005 vs. 2003	Patient deprivation Most vs. Medium vs. Least deprived	Practice size Large vs. Medium vs. Small
**QOF indicators**					

**QOF 1** Smoking status	**-**	**-**	30.04** (4.00-225.24)	**-**	**-**

**QOF 2** Smoking cessation advice	**-**	**-**	21.33 ** (2.33-195.50)	**-**	**-**

**QOF 3** Record of BP	**-**	**-**	**-**	**-**	**-**

**QOF 4** BP < 150/90	**-**	**-**	2.56 *** (1.51-4.34)	**-**	**-**

**Non-QOF indicators (not financially incentivised)**					

**NonQOF 1** Lifestyle advice	**-**	**-**	1.98** (1.22-3.18)	1.34 * (1.00-1.79)	**-**

**NonQOF 2** PVD	0.43 * (0.22-0.83)	2.37 *** (1.55-3.65)	**-**	1.54 * (1.02-2.35)	0.47 ** (0.28-0.77)

**NonQOF 3** DM	0.50 * (0.30-0.86)	**-**	**-**	**-**	**-**

**NonQOF 4** Hyperlipid-aemea	**-**	**-**	**-**	**-**	**-**

**NonQOF 5** Alcohol consumption	**-**	**-**	1.84** (1.09-3.10)	**-**	**-**

**NonQOF 6** Protein in urine	**-**	1.48** (1.11-1.98)	**-**	**-**	0.69* (0.49-0.98)

**NonQOF 7** creatinine and electrolyte	**-**	**-**	3.18** (1.36-7.39)	**-**	**-**

**NonQOF 8** Blood glucose	**-**	**-**	1.92 * (1.17-3.16)	**-**	**-**

**NonQOF 9** Serum/total cholesterol	**-**	**-**	2.82 ** (1.55-5.12)	**-**	**-**

**NonQOF 10** ECG	**-**	**-**	**-**	1.38 * (1.04-1.82)	**-**

Multivariate analysis logistic regression:

* p < 0.05

**p < 0.01

***p < 0.001

Smoking status results were not included, nil significant

The primary aim of this analysis was to estimate the effect of area deprivation. In addition, we identified a number of significant associations between other characteristics and indicator achievement, which will be summarised here. Men were twice as likely as women to have an assessment of peripheral vascular disease (nonQOF 2) or diabetes (nonQOF 3) (Tables 3 &5). Participants aged over 73 years old were almost four times more likely to be assessed for peripheral vascular disease compared to those under the age of 63 (p < 0.001) and they are also more likely to be assessed for presence of protein in urine (Tables 3 &5). As reported previously, nearly all indicators improved over time, with greater achievements in incentivised indicators (13). Large practices were less likely to assess peripheral vascular disease (odds ratio 0.47, 95% confidence interval 0.28-0.77) or check protein in urine (0.69, 0.49-0.98) (Table 5). No association was found between the quality of care and patient smoking status.

## Discussion

### Summary of main findings

The achievement of quality indicators for hypertension either did not vary with geographic deprivation, or was higher in patients from more deprived localities. There were few differences in inequalities before and after the implementation of incentives, although there was a general trend for quality to improve over time, and some indicators reached a ceiling of high achievement in all deprivation categories.

Additionally, we found that using patients' postcode-based deprivation scores gave similar results to using practices' area deprivation scores, derived from the weighted mean of relevant IMDs to the postcodes of all practice's registered patients.

### Strengths and limitations of the study

This study has a number of strengths. To the best of our knowledge, this is the first study to assess the relationship between deprivation and non-incentivised indicators of care for hypertension. All the indicators are evidence-based and validated by independent panels including general practitioners (Table 1). Data were collected by hand searching both the electronic and paper patient records using clear criteria. Credit was given to any mention of the care even if it was not fully documented.

There was an adequate response rate of 76%, and the responders were similar with respect to age and sex to non-responders. Therefore, we think it unlikely that response bias has had a substantial affect on the results, although it is possible that there were differences in the level of deprivation between responders and non-responders. The practices in this study were broadly representative of the English national range of the socioeconomic deprivation [14]. Most of the quality indicators in this study referred to processes of the health care rather than outcomes. Process measures have the following advantages over outcome measures for assessing the quality of health care. There are many causes of changes in health status other than health care, and there are many problems in adequately adjusting outcomes for differences in case mix [17]. Processes are also more sensitive measures of quality than outcomes, and more clearly linked to any action that should be taken to improve quality [18]. Moreover, process measures are currently the basis of the Quality and Outcomes Framework and other pay for performance schemes, and therefore are clearly relevant to those schemes and this paper [19,20].

This study assessed patient area deprivation using patient postcodes, rather than practice postcodes which have been used in previous studies [7-9]. Although using patients' postcode-based deprivation scores is a more accurate measure of deprivation than simply using the practice postcode deprivation, the patients' socioeconomic status might vary despite sharing the same postcode. Ideally further studies should evaluate the correlation of quality of care with patient's income and level of education, but this information is difficult to obtain.

Since 2004 the GP contract has been reviewed, and in 2006 the two hypertension indicators related to smoking were amalgamated with other smoking indicators onto a single smoking domain [19,20].

Limitations include that we assessed recorded care, and it is possible that the care was delivered without being recorded. However, recording is an essential component of quality in a team-based approach to chronic disease management [21]. The study was also based on a small number of practices and patients, due to the practical constraints of collecting data manually from patient records. We categorised participants into thirds in terms of area deprivation. We set the IMD range of these subgroups to give almost equal numbers in each group rather than comparative to the national IMD range. This is because the analysis was set to evaluate the health inequalities within the sample. Only three bands of deprivation were compared. As there is a chance that the differences in quality lie in the bottom quintile, not the bottom tertile, we repeated the analysis using different break points for deprivation, with no substantial changes to the results, and so are confident that our results are not driven by the methods chosen. Nevertheless, a study which focuses on more extreme deprivation may find different results, and this would be an interesting area for further research.

We did not assess the effect of severity of disease, and all patients with hypertension who met our inclusion criteria were eligible. The ethnicity of the patients was not considered in the analysis, as 95-97% of the Norfolk population is classified as 'White British' [22].

Whilst we set out to investigate the relationship between deprivation and quality, our secondary analyses included approximately 70 other univariate analyses comparing baseline characteristics of patients with our varied quality indicators. We used a conventional p-value of < 0.05 to determine statistical significance, but could have used a p-value of p < 0.01 or less in view of the multiple statistical testing. This would have produced a less nuanced but more consistent result that deprivation had no significant effect on achievement of any of the indicators.

### Comparison with existing literature

Our findings are consistent with those reported by Strong et al, who assessed quality of care in 38 general practices. They found either no association between indicator achievement and deprivation, or better care in more deprived areas [9]. Equally, 8,515 practices were assessed by Ashworth et al [8], who reported slightly lower achievement of incentivised indicators for hypertension in 2004/5 in the most deprived areas compared to the least deprived areas, with the near disappearance of this gap by 2006/7.

## Conclusion

This study showed that participants from more deprived areas received at least equivalent, and sometimes higher quality care for hypertension than those from less deprived areas. This differs from previous research which has reported the expected poorer care for more deprived populations, but agrees with other papers which have found better care in more deprived areas [9,23]. Further research using accurate measures of individual deprivation on a larger population is needed to explain the different findings in different studies.

## Ethics approval

The Norwich Research Ethics Committee provided ethical approval of the study (REC 05/Q0101/37). All participants gave informed consent.

## Competing interests

The authors declare that they have no competing interests.

## Authors' contributions

NS designed the study. SH made the statistical analysis with the help of NS and RH. SH, NS and RH wrote the article. All authors have read and approved the final manuscript.

## Pre-publication history

The pre-publication history for this paper can be accessed here:

http://www.biomedcentral.com/1472-6963/11/297/prepub
